# Occurrence, antibiotic resistance profiles and associated risk factors of *Klebsiella pneumoniae* in poultry farms in selected districts of Somalia Reginal State, Ethiopia

**DOI:** 10.1186/s12866-024-03298-1

**Published:** 2024-04-24

**Authors:** Mohamed Abdi Kahin, Abdimalik Hussein mohamed, Ahmed Abdi Mohomed, Mubarik Ali Hassan, Haben Fesseha Gebremeskel, Isayas Asefa Kebede

**Affiliations:** 1 College of Veterinary Medicine, JigJiga University, JigJiga, Ethiopia; 2 Ministry of Livestock and Rural Development, Hargeisa, Somaliland; 3 School of Veterinary Medicine, Wolaita Sodo University, P. O. Box 138, Wolaita Sodo, Ethiopia; 4 School of Veterinary Medicine, Ambo University, P. O. Box 19, Guder, Ethiopia

**Keywords:** Antimicrobial sensitivity, Chicken, Drug resistance, Klebsiella pneumoniae

## Abstract

**Background:**

*Klebsiella pneumoniae* is an opportunistic infection that causes production losses and death in the chicken industry. A cross-sectional study was conducted on exotic chicken breeds reared at the Jigjiga poultry farm from November 2022 to May 2023 to estimate the occurrence, associated risk factors, and antimicrobial susceptibility profiles of *Klebsiella pneumoniae*. The chickens were selected using systematic random sampling techniques. A total of 384 cloacal swabs were collected aseptically and transported to the laboratory for analysis. For statistical analysis, STATA® version 14.0 statistical software was used.

**Results:**

From 384 examined faecal samples, 258 (67.2%) prevalences of *Klebsiella pneumoniae* were found. Furthermore, the association of the study’s risk factors with the prevalence of *Klebsiella pneumoniae* was explored, and no statistically significant association was identified between sex and age. Nonetheless, relative prevalence at the age level was higher in chickens aged 12 months (67.6%) and Sasso breeds (90.0%). Similarly, male chickens and those raised for meat and egg production had a high prevalence rate of 72.5 and 75.8%, respectively. A total of 30 isolated *Klebsiella pneumoniae colonies* were tested in vitro for antibiotic sensitivity for six drugs, and it was shown that *Klebsiella pneumoniae* is moderately sensitive to Penicillin G (43.3%) while having higher resistance to Oxytetracycline (80.0%).

**Conclusions:**

The current findings revealed that the research area had the highest prevalence of *Klebsiella pneumoniae*, and the isolates were resistant to commonly used drugs in the study area. Thus, a long-term intervention plan, thorough research to determine a nationwide status, as well as further multi-drug resistance patterns and molecular characterization, were urged.

## Introduction

Ethiopia has one of Africa’s greatest livestock populations [1]. Livestock is important for generating revenue, offering job opportunities, maintaining food security, providing services, adding assets, social, cultural, and environmental values, and sustaining livelihoods [2]. Poultry is one of the world’s livestock, with an estimated 16.2 billion people, 71.6% of whom reside in developing countries, producing 67,718,544 metric tons of chicken meat and 57,861,747 metric tons of hen eggs every year [3].

The poultry industry provides a chance to feed the world’s rapidly rising population while also providing revenue to resource-limited farmers [4]. Furthermore, in many parts of the modern world, poultry is regarded as the primary source of not just inexpensive animal protein but also high-quality human food. The primary goal of chicken rearing in all production systems is egg and meat production for income creation and home consumption. Animal agriculture in developing nations is under intense pressure to meet the need for animal protein caused by the ongoing expansion of the human population, as well as to have a surplus for international commerce [5].

In Africa, village poultry accounts for more than 70% of chicken products and 20% of animal protein intake. Over 80% of the human population in East Africa lives in rural settings, and over 75% of these households retain indigenous hens, with Ethiopia being no exception [6]. Ethiopia has an estimated poultry population of 60.5 million [7]. The poultry industry is characterized by tiny scavenging flocks of local hens and a few farms in the commercial subsector with various flock sizes [8]. Small and medium-scale producers account for the majority of commercial poultry production in Ethiopia [9]. In Ethiopia, 99% of hens are raised in traditional backyard systems, whereas 1% are raised in intensive management systems [10].

The poultry sector is the fastest-growing of the animal-producing industries. As a result, the production of poultry protein products has recently increased significantly in many of these countries. Nonetheless, it has been hampered by several restrictions. Among the restraints, poultry diseases continue to be a key impediment to its expansion. Several pathogens, including viruses, bacteria, and fungi, can cause disease in chickens [11]. Environmental variables may contribute to the clinically recognized symptoms and lesions caused by these infections [12]. Bacterial infections in the respiratory system are extremely important in chicken production, accounting for around 30% of mortality each year [13].

*Klebsiella bacterium* is a gram-negative, rod-shaped, non-motile, encapsulated opportunistic pathogen. This bacterium belongs to the Enterobacteriaceae family, is anaerobic facultative, is 1.0 mm in length has a mucoid colony, and is responsible for several diseases in both animals and humans [14]. It is the second most common species of Enterobacteriaceae and is found on the mucosal surfaces of animals, including dogs and humans, as well as in soil, water, and food environments [15]. These bacteria often test positive for the Voges-Proskauer test and generate lysine decarboxylase but not ornithine decarboxylase. *Klebsiella spp.* is found in the mucosa of the upper respiratory, gastrointestinal, and urogenital tracts of humans and animals, and causes pneumonia, nasal infection, urinary tract infection, and biogenic infection in men. *Klebsiella pneumoniae* and *K. oxytoca* are opportunistic pathogens associated with severe nosocomial infections such as septicemia, pneumonia, and urinary tract infections in birds [16].

*Klebsiella pneumoniae* causes substantial septicemia, urinary tract infections, pneumonia, and soft tissue infections, particularly in immunocompromised hosts such as young chickens [17]. Some factors for *Klebsiella* pathogenicity have been proposed, including capsular antigen (the most important virulence factor in *Klebsiella pneumoniae*), adhesives, lipopolysaccharides, and siderophores [18]. *Klebsiella pneumoniae*, in addition to nosocomial infection, spreads through foodstuffs and is hence frequently classified as a foodborne disease agent. *Klebsiella pneumoniae* has been identified in shellfish, frozen food, and fresh chicken meat [19]. Moreover, considering the possibility of food-borne infections spreading to humans through the food chain and the advent of super-resistant bacteria [19], *Klebsiella pneumoniae* is a well-known zoonotic conditional pathogen of the Enterobacteriaceae family [20]. Its multidrug resistance (MDR) is concerning.

In chicken farming, antibiotics are often utilized as prophylactics, growth promoters, and medications [21–23]. It has been documented that chicken manure, goods, and the overall environment contain antibiotic-resistant bacteria [24–26]. Consumption of chicken carcasses or eggs may upset the normal balance of bacteria in the human digestive tract since these bacteria are resistant to antibiotics and are commonly found in the digestive systems of poultry. The release of chicken byproducts into the environment, poor sanitation, and bacterial persistence in the environment can all be considered contributing elements to the emergence of drug-resistant variation strains [21].

Nonetheless, there is a serious risk to international health because of the rise of antibiotic-resistant bacterial strains in the food chain [23, 27]. Antibiotic-resistant bacteria could proliferate even in the presence of antibiotics. Further exacerbating this problem is the widespread dissemination of resistance genes across many bacterial species in the digestive tract via plasmids [23–25]. Antibiotic resistance has also been shown to be significantly influenced by the transmission of resistance plasmids across unrelated bacteria [23–25].

Since *Klebsiella pneumoniae* is an opportunistic infection that can harm both people and poultry, this study aimed to evaluate the prevalence and pattern of antibiotic resistance of this bacteria in chicken feces. Moreover, the occurrence of *Klebsiella pneumoniae* in chickens in Ethiopia is poorly reported. Mainly, data are scarce on the prevalence and antimicrobial susceptibility test of *Klebsiella pneumoniae* in the research area. Thus, the current study’s objectives were to estimate the occurrence, associated risk factors, and antimicrobial susceptibility profiles of *Klebsiella pneumoniae* in chickens in Jigjiga town, Somalia regional state, Ethiopia.

## Materials and methods

### Description of the study area

**Fig. 1:**
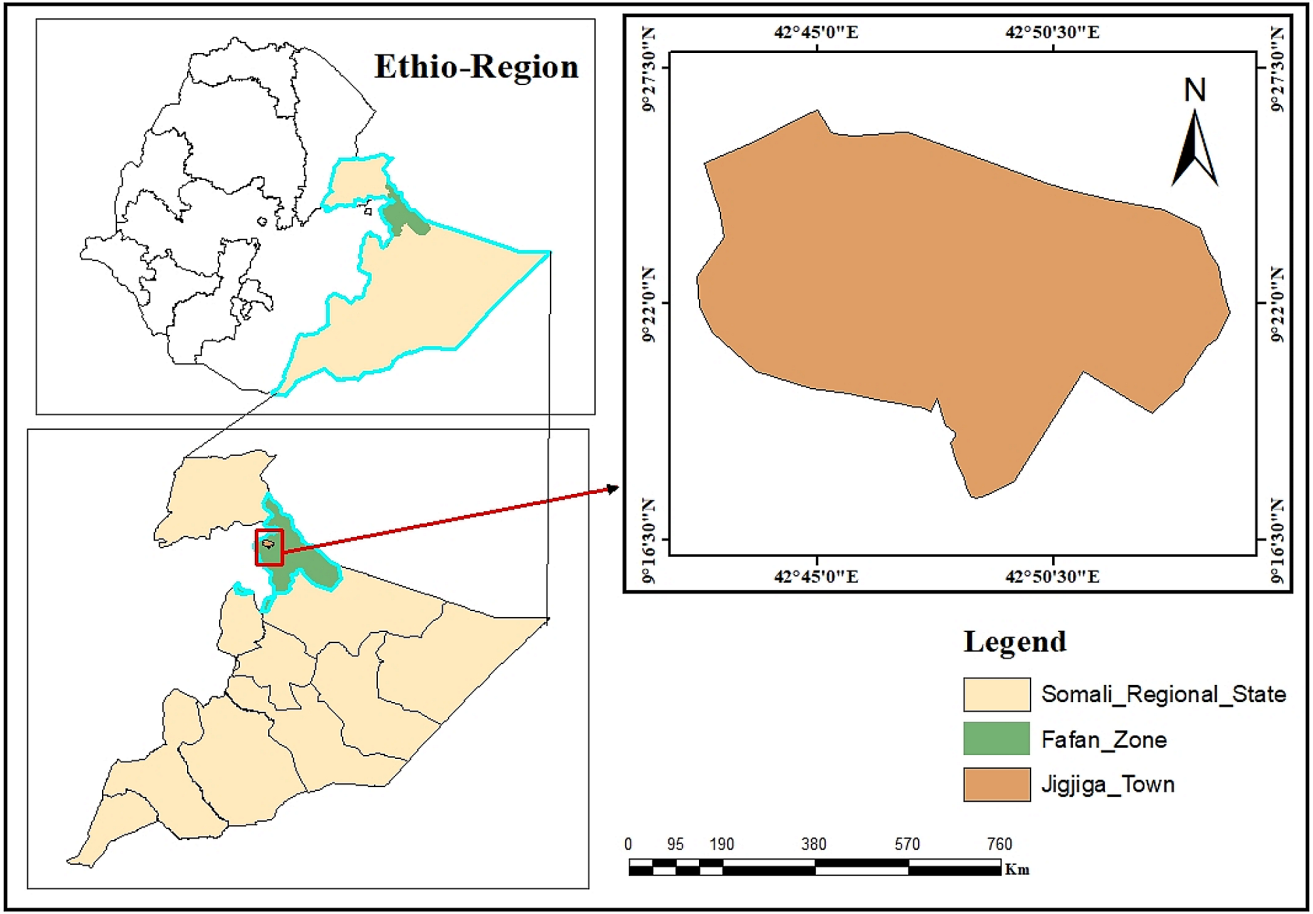
Map of the study area. (Source: ArcGIS, 2024)

The research was carried out in and around Jigjiga, Somali Regional state, eastern Ethiopia (Fig. 1). Jigjiga, also known as the “Fafan Zone” currently, is one of the SRSE’s nine administrative zones. Jigjiga City, the zonal and regional capital, lies 630 km southeast of Addis Ababa. The zone is located in the northern section of SRSE and shares boundaries with the Republic of Somalia in the east, the Oromia regional state, the Fik zone in the west, and the Degahbour zone in the south. The total land cover is 40,861 km^2^, with rangeland covering 36,629 km^2^ [28]. About 52.6%, 31%, and 7% of the zone’s topography can be classified as flat to mild slopes, hills, and steep slopes, respectively. The temperature in the area is normally high all year, with the mean lowest and highest values of roughly 20^o^C and 35^o^C, respectively. The average annual rainfall is 660 mm and is bimodal. The region’s cattle population is predicted to be 2,826,700, including 489,000 in the Fafan zone [28]. Pastoralism, agro-pastoralism, and sedentary production methods make up 34.1, 56.8, and 9.1% of the Fafan Zone, respectively [29].

### Study animals

The animals employed in this investigation were healthy chickens of all ages, exotic breeds (Bovans Brown, White leghorn, Sasso), and both sexes reared at the Jigjiga poultry farm under an intensive management method.

### Study design

A cross-sectional study was conducted on exotic chicken breeds reared at the Jigjiga poultry farm from November 2022 to May 2023 to estimate the occurrence, associated risk factors, and antimicrobial susceptibility profiles of *Klebsiella pneumoniae*. The chickens were selected using systematic random sampling techniques.

### Sample size determination

This sample size was determined according to the Thrusfield [30] formula, with a 95% confidence interval and a 5% precision.

$${\rm{n}} = {1.96^2}*{\rm{Pexp }}\left( {1 - {\rm{ Pexp}}} \right)/{{\rm{d}}^2}$$

Where.

n = required sample size.

P_exp_=expected prevalence, 50%.

d = required precision, 5%.

Thus, a total of 384 cloaca swab samples were collected from chickens.

### Sample collection and transportation

Eight registered chicken farms in and around Jigjiga town were purposefully chosen for the study based on the consent of the farm owners. The size of each of the examined farms was similar, with an average of 12 flocks (flock size > 45) of hens housed at a time (all-in, all-out). A sample of live, clinically healthy hens of various ages was taken from a chosen farm proportionally. During sample collection, farm attendants were interviewed and recorded about the features of the chickens (Age, Sex, Breed, and Purpose of production) and management parties (beddings, litter disposal, and new case handling) to establish risk factors associated with the occurrence of *Klebsiella pneumoniae*. Then, the samples were obtained aseptically from chicken cloaca using sterile swab tools. The cloacal swabs were collected and placed in a transport medium (0.1% buffered peptone water (enriched media) in a test tube)) before being transported to the Jigjiga Microbiology Laboratory using an ice box.

### Isolation and identification of *Klebsiella pneumoniae*

To establish a stock solution for the poultry fecal samples, 1 g was combined with 10 ml of deionized water. The stock was then serially diluted tenfold, and 1 ml of each dilution (10^− 2^, 10^− 4^, and 10^− 6^) was dispensed into sterile Petri plates labeled correspondingly. Eosin methylene blue (EMB) (Hardy Diagnostics agar, USA) and MacConkey (MAC) agar (Oxoid, UK) were aseptically distributed and gently swirled into separate aliquots of samples. To stimulate the development of Klebsiella species, the plates were solidified and incubated for 24–48 h at 35–37 ^o^C [14]. Following this, separate colonies were subcultured on freshly prepared MAC, and streaking was repeated to achieve pure Klebsiella species cultures for further study. Klebsiella species colonies show pink or red on MAC agar. *Klebsiella pneumoniae* was identified by a biochemical test (indole, methyl red, motility, Vogeus proskues, citrate utilization, and triple sugar iron test) [31]. *Klebsiella pneumoniae* was recognized as a biochemical test result of fermented sucrose, lactose, and glucose with the generation of acid within 24–48 h of incubation, a negative reaction in the VP test, a positive reaction in the MR, and an indole test [32].

### Antimicrobial test

Thirty isolated *Klebsiella pneumoniae* colonies were evaluated for antibiotic sensitivity on Mueller-Hinton agar (Oxoid, UK) using the Kirby-Bauer technique [33]. All disks used in the disc diffusion test were acquired from Oxoid England in the following concentrations: oxytetracycline (30 mcg), sulphamethoxazole (25 mcg), nalidixic acid (30 mcg), cefaxime (5 mcg), tetracycline (30 mcg), and penicillin G (30 mcg). The cultural turbidity was corrected to the 0.5 MacFarland standard. The sterile cotton swab was dipped in the suspension and spread evenly across the Mueller Hinton agar surface [31]. After a few minutes, antibiotic discs were spread on the surface of the infected plate and incubated at 37 °C for 16 h. The micrometer zone of diameter was measured and classified as sensitive, intermediate, or resistant [34, 35].

### Data management

Data collected in the field was recorded and stored in an Excel spreadsheet version 2016. The data was then imported into STATA® version 14.0 statistical software to calculate prevalence. Univariable analysis was performed on all predictors by fitting them into separate logistic regression models to assess their unconditional associations. Finally, the 95% confidence interval (CI) was calculated, and disease-associated risk factors with a *p*-value of less than 0.05 were taken as significant.

## Results

For this study, 384 cloaca swabs were bacteriologically examined to obtain information on the prevalence of *Klebsiella pneumoniae* within farms and its associated risk factors. It was revealed that prevalence at the age level was higher in chickens > 12 months (67.6%), and prevalence at the breed level was highest in Sasso (90.0%) compared to counterparts. Similarly, male chickens and those meant for meat and egg production had a high prevalence rate of 72.5% and 75.8%, respectively (Table 1). Overall, the prevalence was 67.2%.

**Table 1:** Prevalence of *Klebsiella pneumoniae* in the study area

Variables	Categories	No. of examined	No of positive N (%)
Age	< 12 months	111	75 (67.6%)
	> 12 months	273	183 (67.0%)
Breed	Bovans	162	117 (72.2%)
	White leghorn	152	78 (51.3%)
	Sasso	70	63 (90.0%)
Sex	Male	40	29 (72.5%)
	Female	344	229 (66.6%)
Type of production	Egg only	213	131 (61.5%)
	Meat only	51	36 (70.6%)
	Meat and eggs	120	91 (75.8%)
Total		384	258 (67.2%)

Furthermore, the relationship between risk factors and *Klebsiella pneumoniae* occurrence was assessed. However, there was no statistically significant (*p* > 0.05) correlation between risk factors (age and sex) and the prevalence of the bacteria (Table 2).

**Table 2:** The association of risk factors with the occurrence of *Klebsiella pneumoniae*

Variables	Categories	No of positive N (%)	x^2^	*p*-value
Age	< 12 months	75 (67.6%)	0.010	0.918
	> 12 months	183 (67.03%)		
Breed	Bovans	117 (72.2%)	59.86	0.001
	White leghorn	78 (51.3%)		
	Sasso	63 (90.0%)		
Sex	Male	29 (72.5%)	0.572	0.450
	Female	229 (66.6%)		
Type of production	Egg only	131 (61.5%)	7.459	0.024
	Meat only	36 (70.6%)		
	Meat and eggs	91 (75.8%)		

**Fig. 2:**
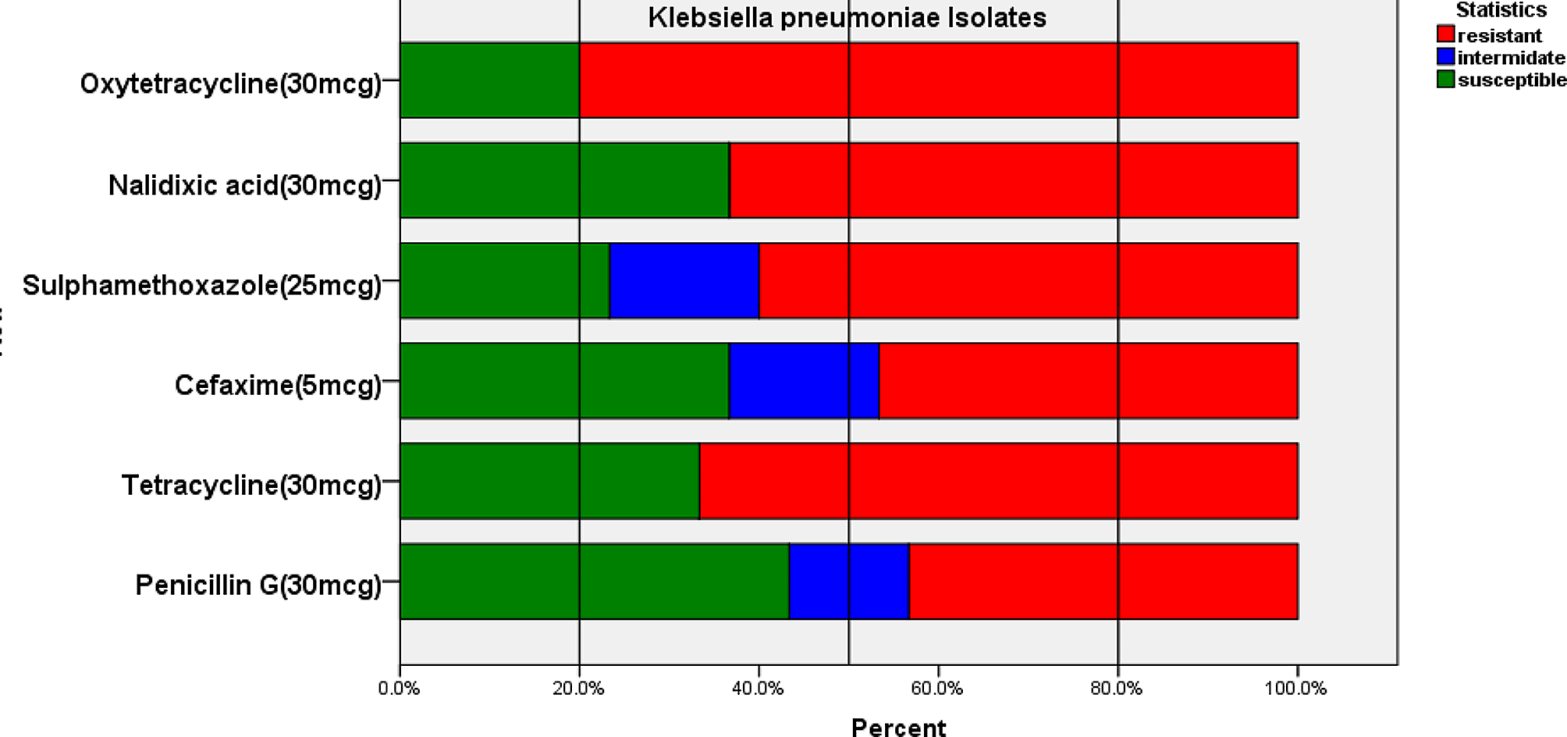
In-vitro antimicrobial susceptibility of the isolated *Klebsiella pneumoniae*

Thirty (*n* = 30) *Klebsiella pneumoniae* isolates were tested for antibiotic susceptibility in vitro and the results were evaluated [35]. Accordingly, isolates developed high resistance to Oxytetracycline (80.0%), followed by Tetracycline (66.7%), Nalidixic acid (63.3%), and Sulpha-methoxazole (60.0%). Conversely, isolates were susceptible to Penicillin G (43.3%), Nalidixic acid (36.7%), and Cefaxime (36.7%). Furthermore, sulphamethoxazole (16.7%), and Cefaxime (16.7%) were recorded as intermediate reactors for the isolates (Fig. 2).

## Discussion

*Klebsiella pneumoniae* is a bacterium that can infect the respiratory system of chickens, resulting in loss of output and mortality [14]. The current study found an overall prevalence of *Klebsiella pneumoniae* of 62.7%, which agrees with the finding of Aly et al. [36], who isolated *Klebsiella* spp. (63%) associated with bile and intestinal content of slaughtered chickens, but significantly higher than the finding of Puspandari et al. [37], who reported an 8.8% prevalence rate from Indonesia. It was shown to be greater than the study conducted in Egypt, which revealed a 10% prevalence of *Klebsiella pneumoniae*, Dashe et al. [38], reported 8.8%, and Popy et al. [39], who reported a 6% prevalence of *Klebsiella* spp. Also, it was higher than the report of Bushen et al. [40], who identified 22.6% of *Klebsiella pneumoniae* from chicken in Ethiopia. The discrepancy in prevalence between the current study and others is most likely owing to chicken farm management practices, sample types (faeces, bile, intestinal contents), and sample sizes. Furthermore, the pattern of antimicrobial use by poultry producers varies significantly: some farms employ antimicrobials as feed additives, which might be ascribed to variable prevalence.

The current study found statistically no relevance between sex and age. Female chicken had a high prevalence of *Klebsiella pneumoniae* relative to sex, chicken for those 12 months relative to age, Sasso relative to breed, and meat and egg-producing chicken relative to production. This variation demonstrates that females, Sasso, meat and egg-producing chickens, and chickens aged 12 months are of interest, implying that they are reservoirs of *Klebsiella pneumoniae*. However, breed and chicken production systems have an association with disease occurrence. Also, the disease was highest in Sasso (90.0%) followed by Bovans (72.2%), and lowest in White leghorn (51.3%) breeds. This might be attributed due to variations in husbandry measures, and disease resistance ability (physiological and immune factors) of the studied breeds.

*Klebsiella pneumoniae* has been documented to be a widely widespread pathogenic agent with a high prevalence of antimicrobial resistance, however antibiotic sensitivity of *Klebsiella pneumoniae* has not been thoroughly researched, particularly in poultry [41]. In vitro, antibiotic susceptibility assays were performed on 30 isolated *Klebsiella pneumoniae* in this study. Figure 2 depicts the percentage of isolates classified as sensitive, intermediate, or resistant to each antimicrobial agent. The study panel includes a total of six antimicrobials and the isolates exhibit strong resistance to Oxytetracycline (80.0%), whereas the intermediate resistance is to Sulphamethoxazole (16.7%).

This finding demonstrated that the highest susceptibility of *Klebsiella pneumoniae* was suggested to Penicillin G (43.3%) because they are not commonly utilized in the study area in veterinary services. This finding was lower than the 96.7% sensitivity to Penicillin G reported by Tefera et al. [42]. This difference from the prior study could be attributed to the level of antibiotic use on the farm.

The current study found that oxytetracycline was highly resistant, which is slightly in line with resistance reported by Bushen et al. [40], and Permatasari et al. [31] who recorded 85.7% and 75%, respectively, but less than the resistance reported by Otalu et al. [43], who recorded 100%. Again, tetracycline was found to have significant resistance (66.7%), which is lower than the 78.8% reported by Bushen et al. [40]. These could be the result of repeated usage of this drug. Tetracycline, for example, was utilized therapeutically, preventively, and to stimulate growth in the current investigation.

Sulphamethoxazole resistance was higher (60.0%), which is inconsistent with the findings of Permatasari et al. [31] who found 28%. This change could be related to repeated therapeutic or indiscriminate antibiotic use. In general, resistance to these medications in gram-negative bacteria may be attributable to the transfer of resistance genes from gram-positive bacteria to -lactamase genes [44].

Antibiotics are extensively used in both humans and animals for therapy and disease management. However, extensive usage of antibiotics may contribute to the growth of antibiotic resistance. The introduction of antimicrobial-resistant (AMR) bacterial strains regularly threatens the effective treatment of numerous infectious illnesses [45]. The widespread use of antibiotics in animals for treatment, growth promotion, and disease prevention causes high selection pressure among microbial agents, which may contribute to the emergence of drug- and multidrug-resistant bacteria and put human health at risk of becoming infected with these transferred zoonotic-resistant bacteria.

The crisis of drug and multi-drug resistance has jeopardized existing and future enhanced modern therapeutic efficacy in animals and people [46]. For example, the introduction of AMR from poultry and poultry farms has been identified as a possible community health risk since it can be transferred through food chains and direct contact with chicken and poultry products [47, 48]. There are considerable gaps in combating infectious diseases, particularly in low and middle-income countries like Ethiopia, due to the high frequency of infection and inappropriate use of antibiotics in livestock [40]. Thus, antibiotic resistance may arise from improper drug usage at poultry farms and the nature of *Klebsiella pneumoniae* which are ubiquitous.

In our findings, *Klebsiella pneumoniae* showed substantial resistance to the majority of the antimicrobials evaluated, which is quite concerning. A previous study on *Klebsiella pneumoniae* found that uncontrolled antimicrobial consumption, inappropriate antibiotic prescription, substandard drug quality, and a lack of effective nosocomial infection prevention measures were key factors in the spread of antimicrobial resistance across studied countries [48, 49]. Because these circumstances may exist in Ethiopia, the increased medication resistance identified in the current investigation might be attributed to these variables.

### Limitation of the study

The current study does not include molecular characterizations and identifications of other bacteria due to the low financial sources, and short study period.

## Conclusion

The current study revealed that *Klebsiella pneumoniae* was prevalent in the study area. Also, an in-vitro antibiotic sensitivity test revealed high sensitivity to Penicillin G and high resistance to oxytetracycline. Breed and chicken production systems have an association with the disease occurrence and the disease was highest in Sasso (90.0%) and lowest in White leghorn (51.3%) breeds. This shows that the disease could be a threat to the poultry farm, which could result in significant economic loss to the farm. As a result, more research into its multi-drug resistance patterns and molecular characterization is required, and farming biosecurity measures, close monitoring of antibiotic resistance, and controlled use of antibiotics should be encouraged.

## Data Availability

All the datasets generated or analyzed during this study are included in this manuscript.
